# Sex differences in borderline personality disorder: A scoping review

**DOI:** 10.1371/journal.pone.0279015

**Published:** 2022-12-30

**Authors:** Xinyu Qian, Michelle L. Townsend, Wan Jie Tan, Brin F. S. Grenyer

**Affiliations:** 1 School of Psychology, University of Wollongong, Wollongong, New South Wales, Australia; 2 Illawarra Health and Medical Research Institute, University of Wollongong, Wollongong, New South Wales, Australia; Medical University of Vienna, AUSTRIA

## Abstract

Borderline Personality Disorder (BPD) is often perceived to be a female-predominant disorder in both research and clinical contexts. Although there is growing recognition of possible sex differences, the current literature remains fragmented and inconclusive. This scoping review aimed to synthesize available research evidence on potential sex differences in BPD. PsycINFO, PubMed, Scopus and Web-of-Science were searched from January 1982 to July 2022 surrounding the key concepts of sex and BPD. Data searching and screening processes followed the Joanna Briggs Institute methodology involving two independent reviewers, and a third reviewer if necessary, and identified 118 papers. Data regarding BPD symptoms, comorbid disorders, developmental factors, biological markers, and treatment were extracted. Data was summarized using the vote counting method or narrative synthesis depending on the availability of literature. Males with BPD were more likely to present externalizing symptoms (e.g., aggressiveness) and comorbid disorders (e.g., substance use), while females with BPD were more likely to present internalizing symptoms (e.g., affective instability) and comorbid disorders (e.g., mood and eating disorders). This review also revealed that substantially more research attention has been given to overall sex differences in baseline BPD symptoms and comorbid disorders. In contrast, there is a dearth of sex-related research pertaining to treatment outcomes, developmental factors, and possible biological markers of BPD. The present scoping review synthesized current studies on sex differences in BPD, with males more likely to present with externalizing symptoms in contrast to females. However, how this might change the prognosis of the disorder or lead to modifications of treatment has not been investigated. Most studies were conducted on western populations, mainly North American (55%) or European (33%), and there is a need for future research to also take into consideration genetic, cultural, and environmental concomitants. As the biological construct of ‘sex’ was employed in the present review, future research could also investigate the social construct ‘gender’. Longitudinal research designs are needed to understand any longer-term sex influence on the course of the disorder.

## Introduction

Borderline personality disorder (BPD) is a complex mental health disorder. It is marked by a pervasive pattern of unstable interpersonal relationships, identity disturbances, affective instability, and impulsive and self-damaging behaviours [1]. Epidemiological studies have estimated that 0.5% to 5.9% of the adult population in the world have BPD [2–4].

Since the introduction of personality disorders in the Diagnostic and Statistical Manual of Mental Disorders (DSM) in 1980’s, BPD has been viewed by clinicians as a female-specific disorder [5]. The DSM-5, for example, indicated that approximately 75% of individuals diagnosed with BPD are females [1]. As a result, the original studies of BPD treatment have focused mainly on females [6]. The field has developed a database of evidence that has largely not included the male experience. More recent research, however, suggest that prevalence rates are not significantly different between males and females [7, 8]. Despite this, BPD is still often perceived to be a female-predominant disorder in both research and clinical contexts.

Due to the lack of conclusive findings, current literature on sex differences in BPD remains fragmented and unclear. For example, findings from an earlier literature review that investigated sex difference across personality disorders was inconclusive [9]. This poses a significant challenge with potential repercussions on treatment applicability and efficacy, especially for males with BPD. Many studies of BPD therapies have primarily been conducted with female participants. For example, the original studies on Dialectical Behaviour Therapy (DBT), one of the most empirically supported treatment for BPD, were female-only [6]. Presently, a handful of studies have examined the effectiveness of DBT for males with BPD [10]. A recent scoping review suggests the relationship between withdrawal behaviours in men with BPD and low utilization of treatment services [11]. This further highlights the need to investigate and develop specific intervention for this group.

Consolidating data on sex and BPD could provide greater insight to treatment effectiveness and guide future research and clinical directions. As such, a scoping review is required to advance the understanding of sex differences in BPD by providing an overview and synthesis of the available research evidence on potential sex differences in BPD, and to identify potential knowledge gaps in literature. As this scoping review was limited by the previous classifications in the literature, it is more accurate to use the biological term ’sex’ instead of the social construct ’gender’ which is rarely reported and not the focus of this review. Specifically, the present scoping review aimed to answer the research question: What are the sex differences and/or similarities of individuals diagnosed with BPD in relation to symptoms, comorbid disorders, developmental factors, biological markers and treatment?

## Methods

### Search strategy

The review was conducted systematically using the Joanna Briggs Institute (JBI) guidelines for Scoping Reviews [12] and the Preferred Reporting Items for Systematic Reviews and Meta-analyses extension for scoping review (PRISMA-ScR) checklist [13]. As the intention of this scoping review was to synthesize the literature on sex differences in BPD, all relevant literature were included regardless of methodology quality to provide a more thorough overview of the topic [14]. Prior to conducting the scoping review, a preliminary search was conducted in Medline, the Cochrane Database of Systematic Reviews, and JBI Evidence Synthesis to ensure that no current or ongoing systematic reviews or scoping reviews on the topic were identified. A librarian at the University of Wollongong supported the research team in the development of the search strategy involving sex and BPD. Primary research studies that examined sex specific results in mixed sex samples were considered for this scoping review. As the aim of the study was to investigate sex differences, studies that recruited transgender participants were excluded due to the complexity in classification. Participants in these studies had a primary diagnosis of BPD as defined either by a classification system (any versions of the DSM or the International Classification of Diseases [ICD]), a validated structured clinical interview, or by a health practitioner. The search strategy included key terms regarding five main areas of interest including, BPD symptoms, comorbid disorders, developmental factors, biological markers, and treatment. Literature search was conducted in PubMed, PsycINFO, Scopus and Web of Science from their date of inception to 2 July 2022. All search strategies used are included in S1 File.

### Study selection

All studies were exported to a referencing software (Clarivate Analytics, PA, United States of America [USA]) where duplicates were removed. The remaining papers were then uploaded to the Covidence review platform, an online web application for screening systematic reviews [15]. This was to facilitate the screening and full-text review processes. The titles and abstracts of the papers were screened independently by two reviewers (X.Q. and W.J.T.). Papers were considered in the present scoping review if they: 1) explored potential sex differences in the population of interest, 2) were retrospective, cross-sectional or prospective primary research studies, 3) were published in English. Studies were excluded if: 1) participants were diagnosed with BPD and another personality disorder, or 2) they were either case reports, letters to editor, protocols, book chapters, conference abstracts, qualitative research, or review study designs. There was no age-based restriction for the included studies. If the relevance of a study was unclear from the title and abstract, then a full-text review was warranted. Differences in opinions between the reviewers were resolved through team discussions until consensus was reached.

### Data extraction and summary

Data was extracted independently by two reviewers (X.Q. and W.J.T.). Extracted data were classified into five overarching categories including symptoms, comorbid disorders, developmental factors, biological markers, and treatment. Each main category was then further broken down into more specific subcategories. For the subcategory of symptomatology, any finding related to the nine DSM-5 symptoms was recorded. For the subcategory of comorbid disorders, any finding of comorbid Axis 1 and Axis 2 disorders were recorded. For the subcategories of developmental, biological, and treatment, any reported differences were recorded. The full table is presented in the data extraction form (S1 Table). Other information such as countries in which the studies took place, population size, and demographic information were also extracted whenever possible. Depending on the population that was recruited in each study, the extracted data was classified into two groups: BPD mixed-sex (i.e., male and female comparisons), and BPD same-sex (i.e., male-only or female-only comparisons).

Data is primarily summarized using two methods. The first method was the use of vote counting to synthesize results in this review [16, 17]. The vote counting method is appropriate for heterogeneous studies and used in recent published research [18, 19]. In mixed-sex comparisons, when a significant difference towards a particular sex was reported, it was quantified as ‘1’ for the respective sex (e.g., male) and category (e.g., impulsivity). The numeric scores were then summed up for each sex, and the difference between the sexes were calculated for each subcategory. This method of vote counting is often used in scoping reviews to investigate the prevalence of a phenomenon [20], or the prevalence of specific phenomenon on binary variables [21–23]. While most previous research only reported vote count in terms of percentage, implementing a set of rules relevant to the number of studies found in the literature search could serve as a way to synthesize results. For this reason, the following rules were predetermined after the number of included studies were determined, but before data extraction commenced. If the number of studies that reported sex differences is smaller than 2, there is neutral evidence. If the difference is between 2 and 3, there is slight evidence for a particular sex. If the difference is 4 to 5, there is some evidence for a particular sex. If the difference is greater than 5, there is substantial evidence for a particular sex. Data from studies that were conducted with only a single sex was recorded but not compared with studies which included both sexes. This was to ensure that the results were not biased due to more studies being conducted with a certain sex.

The second method of summarizing the results was through a narrative synthesis approach. This approach is used when there is insufficient data, i.e., fewer than three studies per researched category. This method is appropriate when no other method of comparison is feasible [24].

## Results

### Descriptive statistics

**Fig 1:**
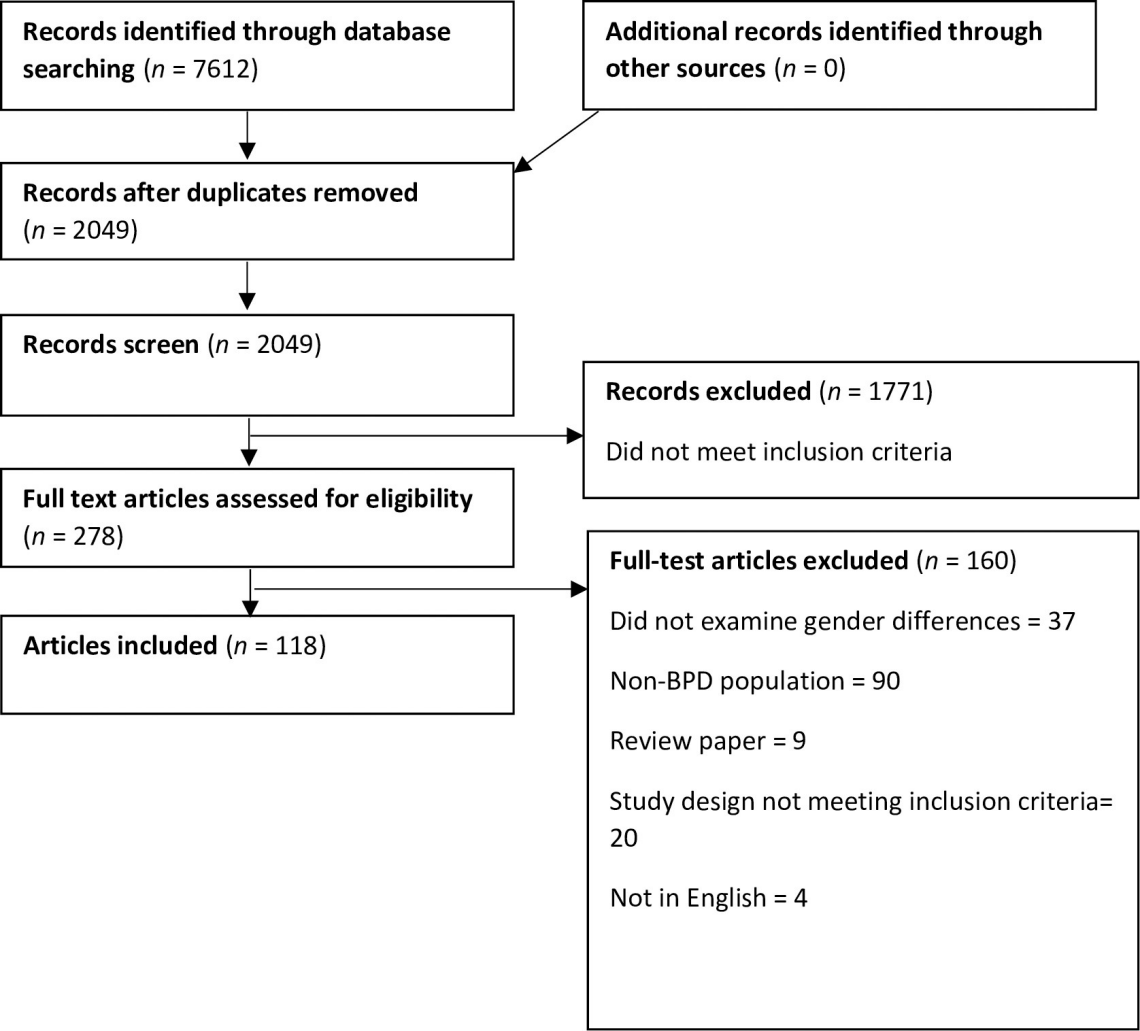
Search results, study selection and inclusion process.

Database searches in July 2022 located 7,612 records, with 2,049 remaining after duplicates were removed. The titles and abstracts of the papers were screened, and 1,771 papers were removed as they did not meet the inclusion criteria. The remaining 278 papers were retrieved for full-text review, where 83 papers were excluded. Reasons for the exclusion are presented according to the PRISMA flow chart [25] in Fig 1. The final 118 papers were included in the scoping review [10, 26–142].

Studies included in the scoping review were published between 1982 and July 2022. Papers published in the last decade (between 2013 and July 2022) made up almost 30.0% of all included studies, with 35 papers published in the last five years (S1 Fig). Among the 118 included studies, the region with the greatest number of published papers that examined sex differences in BPD were in Europe (*n* = 56), with the majority from Germany (*n* = 22). Nearly half of the studies were from the Americas (*n* = 52), with 45 studies from the USA alone. Relatively fewer studies were conducted in Asia (*n* = 5), Oceania (*n* = 3), Middle East (*n* = 1), or of mixed samples (*n* = 1). The full description of the included studies is presented in the study characteristics tables in S2 Table.

### Symptoms and comorbid disorders

Among mixed-sex comparisons on BPD, there was substantial evidence that males tend to display more aggressiveness and impulsivity, and some evidence that females tend to display symptoms of affective instability, suicidal or self-harm behaviours, and unstable relationship/s. Aggressiveness [33, 52, 67, 69, 73, 82, 88, 91, 102, 103] and impulsiveness [33, 48, 58, 69, 88, 91, 109] appeared to be the focus of research for males with BPD in single sex studies. There was substantial evidence from mixed-sex studies that males with BPD are more likely to have comorbid substance abuse and cluster A and B personality disorders than females. Comorbid substance abuse disorder for males with BPD also appeared to be the focus of research in single sex studies [26, 30, 35, 51, 81, 97, 111, 119, 141]. In contrast, mixed-sex studies have displayed substantial evidence for females with BPD to have comorbid anxiety and eating disorders, while some evidence was found for females with BPD to have greater likelihood of mood disorders compared to males with BPD. The full description of the subcategories of BPD symptoms and comorbid disorders in mixed-sex comparison is shown in Table 1.

**Table 1:** Mixed-sex comparisons for BPD symptoms, comorbid disorders, and developmental factors in BPD populations (*n* = 37).

Variable	Strength of evidence^a^	Evidence for	Studies with significant difference towards males		Studies with significant difference towards females	
			*n*	References	*n*	References
**Symptoms**						
Aggressiveness	3	Male	8	[53, 67, 69, 87, 88, 95, 100, 140]	1	[89]
Impulsivity	3	Male	8	[31, 53, 54, 69, 84, 87, 88, 95]	2	[26, 116]
Affective instability	2	Female	0	-	5	[54, 68, 87, 100, 106]
Suicidal/self-harm	2	Female	1	[69]	5	[66, 87, 105, 137, 139]
Unstable relationship	2	Female	1	[66]	4	[26, 69, 78, 87, 89]
Identity disturbance	1	Female	0	-	3	[56, 66, 87]
Dissociation	1	Female	1	[54]	4	[26, 60, 68, 106]
Fear of abandonment	0	Female	1	[69]	1	[87]
Emptiness	0	Female	0	-	1	[54]
**Comorbid disorders**						
Mood	2	Female	0	-	5	[26, 68, 87, 89, 100]
Anxiety	3	Female	0	-	8	[29, 49, 56, 68, 89, 100, 106, 113]
Alcohol and substance	3	Male	15	[30, 31, 44, 49, 56, 67, 70, 88, 95, 100, 105, 106, 113, 120, 133]	0	-
Eating disorder	3	Female	0	-	11	[26, 29, 56, 67, 68, 87, 89, 100, 113, 120, 133]
Cluster A	3	Male	6	[29, 41, 49, 56, 88, 114]	0	-
Cluster B (non-BPD)	3	Male	15	[29, 32, 45, 56, 66–68, 88, 89, 100, 105, 110, 114, 120, 133]	2	[26, 68]
Cluster C	0	Female	2	[110, 114]	3	[31, 32, 88]
**Developmental factors**						
Child maltreatment	1	Female	0	-	2	[60, 115]

*Note*. ^a^Strength of 3 if difference between the number of reports between sexes is > 5, indicating substantial evidence of sex difference. Strength of 2 if difference between the number of reports between sexes is 4 or 5, indicating moderate evidence of sex difference. Strength of 1 if difference between the number of reports between sexes is 2 or 3, indicating weak evidence. Strength of 0 if difference between the number of reports between sexes is < 2, indicating no meaningful difference.

### Developmental factors

There was slight evidence indicating that females with BPD experience may be more likely to experience child maltreatment than males with BPD in the mixed-sex comparisons (Table 1). Early abuse in females with BPD was also a focus of research in single sex female studies [28, 36, 38, 39, 47, 94, 107, 123, 132].

It is important to highlight that while BPD symptoms and comorbid disorders are widely reported in current literature, there is still a paucity of information for the other categories on biological markers and treatment. As such, available findings for these categories were subsequently summarized narratively.

### Biological markers

There were 28 papers regarding biological markers of BPD. Mixed-sex comparisons showed that females have greater cerebellum distribution volume [93], higher cortisol awakening responses [79], increased single nucleotide polymorphism (SNP) 3 and 5 frequencies [99], and larger activations in centro-parietal regions [140]. Trait impulsiveness was negatively related to binding potential values in medial frontal cortex for females but not for males [93]. Males with BPD, had higher activation in the certain regions of the brain, including the left-side cluster (comprising the amygdala, hippocampus, precuneus, and temporal lobe), in clusters of the medial prefrontal cortex, and in the right lateral orbitofrontal cortex (LOFC) and dorsolatera prefrontal cortex (dlPFC) [53].

When compared to controls, females with BPD displayed higher levels of testosterone [34], cortisol awakening response [79, 128, 142], SNP 10 frequency [99], and total plasma homocysteine [132]. Alterations of gut microbial composition was also found, where females with BPD have greater Bacteroidetes/Firmicutes-ratio than healthy controls [123]. There was also weaker activation in the left dlPFC and stronger activation in the posterior cluster in the right dlPFC reported in females with BPD [34]. Several studies reported increased grey matter concentration in the left middle frontal gyrus and right anterior cerebellum, but reductions in the several regions of the brain such as the hippocampus, amygdala, frontal gyri, temporal gyri, and medial temporal lobe [92, 107, 118]. Self-reported trait dissociation was found to be positively correlated with stronger resting-state functional connectivity between the left amygdala and right dlPFC [59].

Males with BPD had lower skin conductance reactivity [103], delta- prolactin, peak- prolactin, and area-under-curve-prolactin [95], and SNP 3 frequency than controls [99]. There was also diminished concentration in the right anterior cerebellum [92] and grey matter volume in several regions of the brain, such as the frontal gyri, LOFC, anterior cingulate cortex, and parietal lobe [92, 109]. Aggressiveness and trait anger was associated with the amygdala, antero prefrontal cortex (aPFC), dlPFC, and right posterior thalamus [33, 53, 67]. Compared to healthy controls, salivary cortisol levels increased in males with BPD, but decreased in females after a social stress exposure [55].

### Treatment

Mixed-sex comparisons showed that compared with males, females were more likely to receive psychological treatment, such as DBT and CBT [49, 62, 65, 133], and psychotropic medication, especially antidepressants and anti-anxiety agents [26, 49, 133]. In contrast, males were more likely to be prescribed with antipsychotic medication [75]. Males are also more likely to have their first BPD diagnosis assessed in a Drugs and Alcohol (D&A) clinic [133], referred to D&A rehabilitation [49] or get treatment in a combination of D&A clinics and psychiatric care [133]. Males with BPD were also less likely to find psychotherapy or hospital admissions helpful compared with females [62], and tend to have fewer therapy sessions [133] or drop out of treatment [64, 105].

Among same-sex comparisons, studies reported that females with BPD were more likely to be restrained and separated using time-out strategy, screened for D&A use, readmitted [122], and have longer stays in hospital than controls [46]. Females with BPD were also more likely to take psychoactive and antipsychotic medication [94, 107, 122]. A female-only study found Fluvoxamine to be an effective for treatment of BPD [80], and a male-only study found that Topiramate was associated with better anger processing and control [72]. DBT was found to improve symptoms of BPD in both female- [10, 63, 98] and male-only studies [10, 35]. However, male-only comparisons indicated that patients with BPD were more likely to drop out of treatment compared to controls [104].

## Discussion

The present scoping review provides an overview of available research evidence on sex-related differences in symptoms, comorbid disorders, developmental factors, biological markers, and treatment of BPD. A total of 118 papers were identified and the extracted data highlight several findings. More than half of the reviewed papers were published in the last decade. Among the included studies, literature published in Western countries, specifically the USA, dominated the field. This indicates a potential literature gap regarding the cross-cultural generalisability of findings to non-Western populations. The scoping review also revealed that a large proportion of literature were cross-sectional studies. Although cross-sectional designs are useful for establishing preliminary evidence, they are unable to investigate variable patterns over time and the temporal relations between outcomes and risk factors. This is particularly important when elucidating the long-term course of BPD symptoms or the causal inference of the development of BPD and comorbid disorders, developmental factors, biological markers, or treatment outcomes.

Findings from the present scoping review corroborate previous studies (e.g., [143]) that there is a propensity for males with BPD to present externalising symptoms (i.e., aggressiveness and impulsivity), while females with BPD tend to display internalising symptoms (e.g., affective instability). These differences that emerged are consistent with those found in population and epidemiological studies. Males are more likely to display aggression, and exhibit high novelty seeking [31, 144]. Females, on the other hand, tend to score higher on harm avoidance and lower on novelty seeking [31, 145], and are more prone to express and talk about their emotions [146]. Thus, BPD pathology may magnify usual sex-based distinction. Interestingly, evidence for sex differences in the present review was more substantial for symptoms that are more prevalent in males with BPD (aggression and impulsivity), but moderate to weak for symptoms that are more prevalent in females (affective instability, self-harm, unstable relationship, identity disturbance, and dissociation). This could potentially be explained by the classification and diagnostic criteria for BPD in the DSM. There are comparatively more items for internalizing symptoms than externalizing symptoms, which could have concentrated the scores for externalizing symptoms due to the narrow classification range. A study found that it is more likely for males to be rated as exhibiting symptoms of aggression and impulsivity than females at the same level of BPD liability [69].

Data from the scoping review also suggest that males with BPD have a higher likelihood of exhibiting comorbid substance use disorder while females have comorbid anxiety and eating disorders. This is in line with sex differences in symptomatic manifestation. Males may have a propensity to express distress outwards (i.e., externalization), which is a factor connecting substance use and antisocial behaviour [147]. In contrast, females may have a propensity to express distress inwards (i.e., internalization) which is a factor that characterises mood and anxiety disorders [147]. This sex difference in comorbid disorders, however, could also be due to clinician’s bias regarding sex prevalence of various disorders during diagnosis [5, 148]. For example, with similar symptoms, males are more likely to be diagnosed with anti-social personality disorder while females are more likely to be diagnosed with BPD [5]. Overall, in the present review, the strength of evidence for comorbid disorders is more substantial, apart from mood disorders, compared to symptoms. This could be attributed to the greater number of studies conducted on comorbid symptoms than BPD symptoms included in this review. There are 20 different papers which contributed to 43 counts of evidence for symptoms, while 25 papers contributed to 64 counts of evidence for comorbid disorders. As the research evidence grows, sex differences may be more substantial and more evident.

Despite the growing number of studies examining sex-related differences in BPD, a greater amount of attention was given to BPD symptoms and comorbid disorders. Substantially fewer papers on developmental factors, biological markers, and treatment were identified in the scoping review. This is despite biological markers and psychosocial factors being proposed to potentially contribute to sex differences in symptom manifestation [60]. This posed as a challenge when comparing the findings across studies in these areas due to a dearth of available data. Nonetheless, several noteworthy trends were observed.

Childhood maltreatment or early abuse was more prevalent in females with BPD. This could be explained by differences in the type of abuse reported. Sexual abuse is more often reported in females [149]. In comparison, physical punishment happens more often in males and certain cultures [150], and could be normalized to a certain extent and not perceived and reported as abuse. Furthermore, data could be skewed as males are less prone to report experiences of abuse [151], thus explaining the greater proportion of females with childhood abuse histories.

Females with BPD were also more likely to receive psychological treatment, whereas males were more often referred to D&A rehabilitation. This could be influenced by the difference in symptoms exhibited. As females tend to exhibit internalising symptoms, they are more likely to seek mental health services. Males, in contrast, tend to experience externalising symptoms and therefore may be more likely to seek help for D&A related issues. This form of sample bias in current literature could contribute to the sex bias in BPD prevalence [8]. Although several papers found DBT to be effective in improving symptoms for both females and males with BPD [10, 35, 63, 98], males were less likely to find psychotherapy or hospital admissions helpful [62] and were more likely to drop out [64, 104, 105]. This could be due to males conforming to traditional masculine norms, hence are more reluctant to engage in mental health treatment [152]. It is important to note that available information in these areas is fragmented and scarce. Thus, sex patterns in developmental factors, biological markers and treatment are still inconclusive.

### Limitations

The scoping review methodology has some limitations. Firstly, there is no risk of bias assessment in this review which may lead to biases in the conclusion of this study. Secondly, while the method of vote counting and narrative synthesis are sufficient in providing a broad overview of the literature, the current review is unable to reach a conclusive resolution to all sex related questions in BPD due the relatively small body of literature. Hence, findings from this review raises more questions and highlights the need for further studies, in particular on sex differences in treatment, developmental factors, or biological markers, to fill the gaps in the current literature. As the literature regarding sex differences in BPD grows, further reviews are essential for deeper understanding regarding the topic.

Given the nature of the scoping review, various non-published literature, such as conference abstracts or protocols, were omitted from the content of this paper. Similarly, the papers published in languages other than English were also excluded. Hence, important information in this research area could have been overlooked.

### Future research

With the growth of literature, further systematic reviews and potentially meta-analyses, with risk of bias and quality appraisal assessments are important for the deeper understanding of sex differences in BPD. Apart from the variables investigated in the current review, other moderating factors such as age, population, type of measure, and culture could also be investigated.

Longitudinal research designs could be employed to better understand sex differences in symptoms and comorbid disorders in BPD populations across time. Future research could investigate if sex differences in BPD is simply a product of typical sex-based distinctions also observed in the general population, or if the disorder has an effect on these differences. As the body of research grows, using meta-analysis methods may become feasible. In the present scoping review, the biological construct of ‘sex’ was employed. Future research could also investigate the social construct ‘gender’.

## Conclusion

The present scoping review provides a synthesis of current literature on sex differences in BPD. There is particular focus on symptoms and comorbid disorders, which shows a tendency of males with BPD to more likely exhibit externalizing symptoms and comorbid disorders, while females with BPD to more likely exhibit internalizing symptoms and comorbid disorders. In contrast, there were fewer papers investigating biological markers, environmental factors, and treatment for BPD. Due to the lack of current literature in these areas, results remain inconclusive and more research is required to fill this literature gap.

## Supporting information

S1 File Literature search strategies. (DOCX)

S1 Fig Data of publication of included studies. (TIF)

S1 Table Data extraction form. (XLSX)

S2 Table Study characteristics table. (DOCX)
