# Supplementary material for: Sex differences in borderline personality disorder: A scoping review
Source: PLoS One. 2022 Dec 30;17(12):e0279015. doi: 10.1371/journal.pone.0279015 (PMC9803119; doi:10.1371/journal.pone.0279015)
Supplement: S1 File — (DOCX) [file pone.0279015.s001.docx]

Psycinfo:

http://ezproxy.uow.edu.au/login?url=https://search.ebscohost.com/login.aspx?direct=true&db=psyh&bquery=(((TI+Prevalen*)+OR+(AB+Prevalen*)+OR+(TI+Rat*)+OR+(AB+Rat*)+OR+(TI+Frequency)+OR+(AB+Frequency)+OR+(TI+Percentage)+OR+(AB+Percentage)+OR+(TI+Symptom)+OR+(AB+Symptom)+OR+(TI+Manifest*)+OR+(AB+Manifest*))+OR+((TI+Sign)+OR+(AB+Sign)+OR+(TI+Trait)+OR+(AB+Trait)+OR+(TI+Comorbid)+OR+(AB+Comorbid)+OR+(TI+Co-morbid)+OR+(AB+Co-morbid)+OR+(TI+Morbidity)+OR+(AB+Morbidity)+OR+(TI+Developmental)+OR+(AB+Developmental))+OR+((TI+Cause)+OR+(AB+Cause)+OR+(TI+Aetiology)+OR+(AB+Aetiology)+OR+(TI+Etiology)+OR+(AB+Etiology)+OR+(TI+Bio*)+OR+(AB+Bio*)+OR+(TI+Mortality)+OR+(AB+Mortality)+OR+(TI+Life+expectancy)+OR+(AB+Life+expectancy))+OR+((TI+Outcome)+OR+(AB+Outcome)+OR+(TI+Preference)+OR+(AB+Preference)+OR+(TI+Goal*)+OR+(AB+Goal*)+OR+(TI+Psychotherapy)+OR+(AB+Psychotherapy)+OR+(TI+Therapy)+OR+(AB+Therapy)+OR+(TI+Treatment)+OR+(AB+Treatment))+OR+((TI+Suicid*)+OR+(AB+Suicid*)+OR+(TI+Depress*)+OR+(AB+Depress*)+OR+(TI+Alcohol)+OR+(AB+Alcohol)+OR+(TI+Substance)+OR+(AB+Substance)+OR+(TI+Drug)+OR+(AB+Drug)+OR+(TI+Aggressi*)+OR+(AB+Aggressi*))+OR+((TI+abuse)+OR+(AB+Abuse)+OR+(TI+Sexual)+OR+(AB+Sexual)+OR+(TI+Child*)+OR+(AB+Child*)))+AND+(((TI+gender)+OR+(AB+gender)+OR+(TI+sex)+OR+(AB+sex)+OR+(TI+male)+OR+(AB+male)+OR+(TI+man)+OR+(AB+man)+OR+(TI+men)+OR+(AB+men))+OR+((TI+boy)+OR+(AB+boy)+OR+(TI+girl)+OR+(AB+girl)))+AND+((TI+Borderline+Personality)+OR+(AB+Borderline+Personality)+OR+(TI+bpd)+OR+(AB+bpd))&type=1&searchMode=Standard&site=ehost-live

Pubmed:

(((((((((((((((((((((((((((((((((((Prevalen*[Title/Abstract]) OR Rat*[Title/Abstract]) OR Frequency[Title/Abstract]) OR Percentage[Title/Abstract]) OR Symptom[Title/Abstract]) OR Manifest*[Title/Abstract]) OR Sign[Title/Abstract]) OR trait[Title/Abstract]) OR comorbid[Title/Abstract]) OR co-morbid[Title/Abstract]) OR morbidity[Title/Abstract]) OR developmental[Title/Abstract]) OR cause[Title/Abstract]) OR aetiology[Title/Abstract]) OR etiology[Title/Abstract]) OR bio*[Title/Abstract]) OR mortality[Title/Abstract]) OR life expectancy[Title/Abstract]) OR outcome[Title/Abstract]) OR preference[Title/Abstract]) OR goal*[Title/Abstract]) OR psychotherapy[Title/Abstract]) OR therapy[Title/Abstract]) OR treatment[Title/Abstract]) OR suicid*[Title/Abstract]) OR depress*[Title/Abstract]) OR alcohol[Title/Abstract]) OR substance[Title/Abstract]) OR drug[Title/Abstract]) OR aggresi*[Title/Abstract]) OR abuse[Title/Abstract]) OR sexual[Title/Abstract]) OR child*[Title/Abstract])) AND (((((((gender[Title/Abstract]) OR sex[Title/Abstract]) OR male[Title/Abstract]) OR man[Title/Abstract]) OR men[Title/Abstract]) OR boy[Title/Abstract]) OR girl[Title/Abstract])) AND ((Borderline Personality[Title/Abstract]) OR bpd[Title/Abstract])

Scopus:

(TITLE-ABS ( prevalen* ) OR TITLE-ABS ( rat* ) OR TITLE-ABS ( frequency ) OR TITLE-ABS ( percentage ) OR TITLE-ABS ( symptom ) OR TITLE-ABS ( manifest* ) OR TITLE-ABS ( sign ) OR TITLE-ABS ( trait ) OR TITLE-ABS ( comorbid ) OR TITLE-ABS ( co-morbid ) OR TITLE-ABS ( morbidity ) OR TITLE-ABS ( life AND expectancy ) OR TITLE-ABS ( outcome ) OR TITLE-ABS ( preference ) OR TITLE-ABS ( goal* ) OR TITLE-ABS ( psychotherapy ) OR TITLE-ABS ( therapy ) OR TITLE-ABS ( treatment ) OR TITLE-ABS ( suicid* ) OR TITLE-ABS ( depress* ) OR TITLE-ABS ( alcohol ) OR TITLE-ABS ( substance ) OR TITLE-ABS ( drug ) OR TITLE-ABS ( aggressi* ) OR TITLE-ABS ( abuse ) OR TITLE-ABS ( sexual ) OR TITLE-ABS ( child* ) OR TITLE-ABS ( developmental ) OR TITLE-ABS ( cause ) OR TITLE-ABS ( aetiology ) OR TITLE-ABS ( etiology ) OR TITLE-ABS ( bio* ) OR TITLE-ABS ( mortality ) ) AND (TITLE-ABS ( gender ) OR TITLE-ABS ( sex ) OR TITLE-ABS ( male ) OR TITLE-ABS ( man ) OR TITLE-ABS ( men ) OR TITLE-ABS ( boy ) OR TITLE-ABS ( girl ) ) AND (TITLE-ABS ( borderline  AND personality )  OR  TITLE-ABS ( bpd ) )

Web of science: OR (ti=x AND ab=x)

(Ti=prevalen* or ab=prevalen*) OR (ti=rat* or ab=rat*) OR (ti=frequency or ab=frequency) OR (ti=percentage or ab=percentage) OR (ti=symptom or ab=symptom) OR (ti=manifest* or ab=manifest*) OR (ti=sign or ab=sign) OR (ti=trait or ab=trait) OR (ti=comorbid or ab=comorbid) OR (ti=co-morbid or ab=co-morbid) OR (ti=morbidity or ab=morbidity) OR (ti=(life expectancy) or ab=(life expectancy)) OR (ti=outcome or ab=outcome) OR (ti=preference or ab=preference) OR (ti=goal* or ab=goal*) OR (ti=psychotherapy or ab=psychotherapy) OR (ti=therapy or ab=therapy) OR (ti=treatment or ab=treatment) OR (ti=suicid* or ab=suicid*) OR (ti=depress* or ab=depress*) OR (ti=alcohol or ab=alcohol) OR (ti=substance or ab=substance) OR (ti=drug or ab=drug) OR (ti=aggressi* or ab=aggressi*) OR (ti=abuse or ab=abuse) OR (ti=sexual or ab=sexual) OR (ti=child* or ab=child*) OR (ti=developmental or ab=developmental) OR (ti=cause or ab=cause) OR (ti=aetiology or ab=aetiology) OR (ti=etiology or ab=etiology) OR (ti=bio* or ab=bio*) OR (ti=mortality or ab=mortality)

(ti=gender or ab=gender) OR (ti=sex or ab=sex) OR (ti=male or ab=male) OR (ti=man or ab=man) OR (ti=men or ab=men) OR (ti=boy or ab=boy) OR (ti=girl or ab=girl)

(ti=(borderline personality) or ab=(borderline personality)) OR (ti=bpd AND ab=bpd)
