# Supplementary figures and images for: Sex differences in borderline personality disorder: A scoping review
Source: PLoS One. 2022 Dec 30;17(12):e0279015. doi: 10.1371/journal.pone.0279015 (PMC9803119; doi:10.1371/journal.pone.0279015)

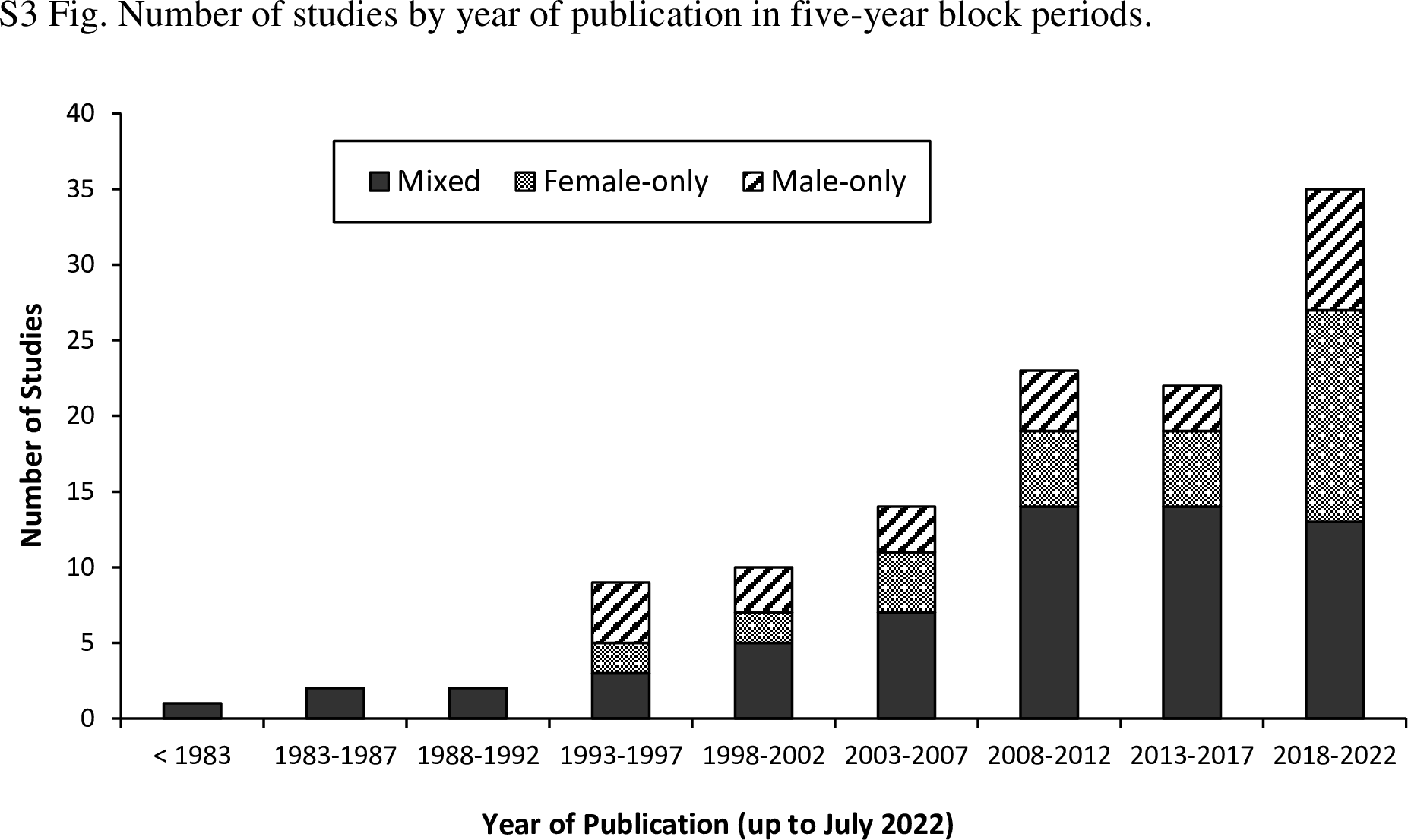

Supplement: S1 Fig — (TIF) [file pone.0279015.s002.tif]
