# Supplementary material for: Sex differences in borderline personality disorder: A scoping review
Source: PLoS One. 2022 Dec 30;17(12):e0279015. doi: 10.1371/journal.pone.0279015 (PMC9803119; doi:10.1371/journal.pone.0279015)
Supplement: S2 Table — (DOCX) [file pone.0279015.s004.docx]

S4 Table. Study characteristics table.

| Title | First author | Country | Year published | Selected sample | | | | | Study Aim / Research questions | Key findings |
| --- | --- | --- | --- | --- | --- | --- | --- | --- | --- | --- |
|  |  |  |  | Population group | Males | Females | Total sample | Mean age |  |  |
| A behavioral comparison of female adolescent inpatients with and without borderline personality disorder | Faulkner, C. J. | USA | 1999 | Clinical | 0 | 35 | 35 | Not stated | Examine the behaviour and inpatient experiences of hospitalized adolescents with and without BPD. | Females with BPD have greater aggression, drug/alcohol use, and longer hospitalization than females without BPD. |
| A prospective, longitudinal, study of men with borderline personality disorder with and without comorbid antisocial personality disorder | Robitaille, M. P. | France | 2017 | Clinical | 37 | 0 | 37 | 33.09 | Further understand BPD, comorbid disorders, health service use, criminality, psychopathic traits, and childhood antecedents, of a community sample of males followed from age 6 to 33 years. | Males with BPD more likely to have suicidal thoughts/attempts, comorbid mood and anxiety disorders, suffer from early emotional disturbance, abuse drug and alcohol, and less likely to be married than males without BPD. |
| A retrospective survey of care provided to patients with borderline personality disorder admitted to a female psychiatric intensive care unit | Gintalaite-Bieliauskiene, K. | UK | 2020 | Clinical | 0 | 29 | 142 | 33 | To describe the management of female patients with BPD admitted to an psychiatric intensive care  unit, Elizabeth Casson House. | Female adolescents report higher borderline symptoms than males. |
| A study of risky sexual behavior, beliefs about sexual behavior, and sexual self-efficacy in adolescent inpatients with and without borderline personality disorder | Penner, F. | USA | 2019 | Clinical | 0 | 50 | 0 | 15.04 | Examine whether adolescent females with BPD differ from inpatient psychiatric controls in regard to: 1) risky sexual behaviours, 2) personal attitudes and perceived peer norms about risky sexual behaviours, and 3) sexual self-efficacy. | Females with BPD had significantly lower self-efficacy to refuse sex, and riskier attitudes and perceived peer norms about sexual behaviour than inpatient controls. |
| A voxel-based morphometric MRI study in men with borderline personality disorder: Preliminary findings | Völlm, B. A. | United Kingdom | 2009 | Clinical | 13 | 0 | 13 | 35.1 | Examine differences in brain volumes between males with BPD and a healthy control group. | BPD in males was associated with impulsivity. BPD group had lower grey matter volumes in superior, middle and medial frontal gyrus, lateral orbitofrontal and inferior frontal cortex, precentral and postcentral gyrus, anterior cingulate, middle temporal gyrus and temporal pole, superior parietal cortex and inferior parietal lobule than healthy controls. |
| Affective interference in borderline personality disorder: The lethality of suicidal behavior predicts functional brain profiles | Soloff, P. H. | USA | 2019 | Clinical | 0 | 23 | 23 | 28.3 | Examine the relationship between medical lethality of suicidal behaviour and neural responses during performance of an affectively modified Continuous Performance Task. | 60.9% of females with BPD had MDD, 73.9% reported a history of childhood abuse, 56.5% reported a history of sexual abuse, 52.5% reported history of psychoactive medication, and 30.4% and 69.6% had attempted high and low lethality suicide. |
| Alterations of the gut microbiota in borderline personality disorder | Rössler, H. | Germany | 2022 | Mixed | 0 | 24 | 45 | 23.75 | To explore the gut microbiota in females diagnosed with BPD. | Bacteroidetes/Firmicutes-ratio (BFR) was larger in BPD compared to HC, suggesting alterations of the gut microbial composition. BPD patients reported more depressive symptoms, dysfunctional eating behaviour and distorted body images, and traumatic events during childhood than HC. |
| Altered emotional decision-making in prisoners with borderline personality disorder | Kirkpatrick, T. | United Kingdom | 2007 | Clinical | 17 | 0 | 17 | 34.65 | Investigate the relationship between BPD, serious offences, and risky behaviour management. | Males with BPD reported higher impulsivity, depressive symptoms, and anxiety symptoms than males without BPD. |
| Altered empathy for psychological and physical pain in borderline personality disorder | Flasbeck, V. | Germany | 2017 | Clinical | 0 | 50 | 50 | 28.7 | Examine empathy for physical and psychological pain from first- and third-person perspectives. | Females with BPD had lower cognitive empathy, higher affective empathy, higher alexithymia, and more traumatic events in childhood than females without BPD. |
| Altered state and trait disgust in borderline personality disorder | Schienle, A. | Austria | 2013 | Clinical | 0 | 30 | 30 | 27.8 | Assess different disgust-related personality traits, as well as visually elicited disgust feelings and the ability to decode facial disgust in BPD. | Females with BPD were more reactive to male faces, reported higher feelings of disgust, and felt less happy when viewing happy pictures than females without BPD. |
| Always on guard: emotion regulation in women with borderline personality disorder compared to nonpatient controls and patients with cluster-C personality disorder | van Zutphen, L. | Netherlands | 2018 | Clinical | 0 | 55 | 55 | 30.8 | Investigate emotional sensitivity and emotion regulation abilities in patients with BPD with a focus on stimulus category specificity and diagnosis specificity. | Females with BPD reported higher dissociation, MDD, and substance abuse, and were more likely to have PSTD, abuse, and neglect than females without BPD. |
| Amygdala and anterior cingulate resting-state functional connectivity in borderline personality disorder patients with a history of interpersonal trauma | Krause-Utz, A. | Germany | 2014 | Clinical | 0 | 20 | 20 | 29.55 | Investigate resting-state functional brain connectivity patterns in un-medicated individuals with BPD compared with age- and education-matched healthy controls. | Females with BPD reported comorbid MDD (40%), substance abuse (20%), panic disorder (10%), social phobia (10%), PTSD (45%), eating disorder (35%). Females with BPD showed a trend towards increased resting-state functional connectivity and diminished negative resting-state functional connectivity compared to controls. |
| Amygdala structure and aggressiveness in borderline personality disorder | Mancke, F. | Germany | 2018 | Clinical | 21 | 37 | 58 | 27.85 | Investigate a sex-mixed sample of BPD patients and healthy volunteers, and to study the alterations of amygdala volume and localized amygdala shape. | Males with BPD more likely to display aggression, abuse substances, and have comorbid antisocial personality disorder. In contrast, females with BPD more likely to have comorbid eating disorders and somatoform disorder. |
| An investigation of differential item functioning across gender of BPD criteria | Sharp, C. | USA | 2014 | Clinical | 45 | 79 | 124 | Not stated | Evaluate gender-based differential item functioning in DSM BPD criteria using item response theory in a large clinical sample of adult psychiatric inpatients. | Males with BPD had higher aggression and impulsivity, and scored lower on BPD criteria. Females with BPD had greater fear of abandonment, unstable relationship, identity disturbances, suicidal behaviour, affective instability, and more likely to have comorbid MDD and eating disorder. |
| An investigation of the relationship between borderline personality disorder and cocaine-related attentional bias following trauma cue exposure: The moderating role of gender | Bardeen, J. R. | USA | 2014 | SUD | 7 | 15 | 22 | 43.09 | Explore the moderating role of gender in cocaine-related attentional biases among cocaine-dependent patients with and without BPD. | Males with BPD exhibited greater attentional bias towards drug-related stimuli than females following exposure to an emotionally distressing cue (post-trauma). |
| An item response theory analysis of the DSM-IV borderline personality disorder criteria in a population-based sample of 11- to 12-year-old children | Michonski, J. D. | United Kingdom | 2013 | Community | 19 | 23 | 42 | Not stated | Evaluate the performance of the DSM criteria for BPD in youth using item response theory in a large, population-based sample of English children aged 11 to 12. Presence of differential item functioning across gender was also evaluated. | Males with BPD more likely to display fear of abandonment, impulsivity, suicidal behaviour, and uncontrolled anger. Females with BPD more likely to experience unstable relationship. |
| Association analysis of SCN9A gene variants with borderline personality disorder | Tadić, A. | Germany | 2008 | Clinical | 49 | 112 | 161 | 33.2 | Test for associations between gene variants of the SCN9A gene and BPD diagnosis as well as particular phenotypes. | Females with BPD higher frequencies of SNP 3 and 5 than males with BPD. |
| Association between history of abuse and borderline personality disorder for hospitalized adolescent girls | Atlas, J. A. | USA | 1995 | Clinical | 0 | 25 | 25 | Not stated | Assess the positive association between histories of physical or sexual abuse as documented in hospital admission records with borderline personality disorder. | Females with BPD more likely to have a history of abuse than females without BPD. |
| Associations between age and cortisol awakening response in patients with borderline personality disorder | Rausch, J. | Germany | 2021 | Mixed | 0 | 54 | 108 | 23.4 | Determine if Cortisol Awakening Response (CAR) in females with BPD is different than females without BPD. | Results not only demonstrated increased CARs in female individuals with BPD compared to HC but demonstrated elevated CARs with increasing age in BPD individuals exclusively. |
| Attention Deficit Hyperactivity Disorder in Prisoners: Increased Substance Use Disorder Severity and Psychiatric Comorbidity | Vélez-Pastrana, M. C. | USA | 2020 | Forensic | 500 | 0 | 500 | 30.66 | Examined whether ADHD was associated with increased severity of comorbid SUD and with increased psychiatric comorbidity among prisoners. | BPD in males associated with ADHD and SUD. |
| Axis I comorbidity of borderline personality disorder | Zanarini, M. C. | USA | 1998 | Clinical | 83 | 296 | 379 | 27.6 | 1) Assess the lifetime occurrence rates of DSM-III-R axis I disorders in borderline personality disorder compared to other personality disorders. 2) Assess the effect of gender on the axis I comorbidity exhibited by borderline patients. | Substance use disorders were more common among male borderline patients, while eating disorders and PTSD were significantly more common among female borderline patients. |
| Axis II comorbidity in borderline personality disorder is influenced by sex, age, and clinical severity | Barrachina, J. | Spain | 2011 | Clinical | 82 | 402 | 484 | 28.3 | Determine the prevalence of concurrent axis II disorders in a large sample of patients with BPD and to investigate the influence of sex, age, and severity on this comorbidity. | Males with BPD more likely to have comorbid antisocial personality disorder than females with BPD. Females with BPD more likely to have comorbid cluster C and dependent personality disorder than males with BPD. |
| Axis II comorbidity of borderline personality disorder | Zanarini, M. C. | USA | 1998 | Clinical | 83 | 296 | 379 | 29.2 | Assess the prevalence of a full range of DSM-III-R axis II disorders in a sample of criteria-defined borderline patients and axis II controls. | Females with BPD more likely to report PD and anxious cluster disorders than females without BPD. Males with BPD more likely to report personality disorders and anxious cluster disorders than males without BPD. Males with BPD are more likely than female borderline patients to have met DSM-III-R criteria for an odd cluster disorder and dramatic cluster disorder. |
| Behavioral and neurophysiological correlates of emotional face processing in borderline personality disorder: are there differences between men and women? | Andermann, M. | Germany | 2022 | Mixed | 21 | 33 | 102 | 27.49 | Investigate how individuals with BPD may interpret facial expressions differently from those without. | Men had lower anger ratings than women and responded slower to angry but not happy faces. The P3/LPP was larger in healthy controls than in individuals with BPD, and larger in women than in men; moreover, women but not men produced enlarged P3/LPP responses to angry vs. happy faces. |
| Borderline personality disorder and prior suicide attempts define a severity gradient among hospitalized adolescent suicide attempters | Aouidad, A. | France | 2020 | Clinical | 51 | 250 | 319 | 14.7 | To investigate the relationship between suicide attempt and BPD symptoms. | Females with BPD symptoms were more likely to attempt suicide than males with BPD symptoms. |
| Borderline personality disorder associates with violent criminality in women: A population based follow-up study of adolescent psychiatric inpatients in Northern Finland | Arola, R. | Finland | 2016 | Clinical | 8 | 25 | 33 | Not stated | Investigate the association of personality disorders assessed in late adolescence and early adulthood to criminal behaviour among a clinical sample of adolescent inpatients treated in psychiatric hospital. | Females with BPD were six times more likely to commit a violent offence. BPD accounted for 78% of all females with personality disorders committing criminal offences and 38.5% in males. |
| Borderline personality disorder in male and female offenders newly committed to prison | Black, D. W. | USA | 2007 | Forensic | 53 | 12 | 65 | 29.5 | Estimate the rate of BPD in male and female offenders newly committed to the Iowa Department of Justice. | The percentage of females meeting the criteria for BPD was more than twice of that for males. |
| Borderline personality disorder: Prevalence and psychiatric comorbidity among male offenders on probation in Sweden | Wetterborg, D. | Sweden | 2015 | Clinical | 11 | 0 | 11 | 35.5 | Estimate the prevalence of BPD in a consecutive sample of adult male offenders on probation or parole in Stockholm, Sweden, and examine comorbidity patterns. | Males with BPD more likely to have comorbid mental disorders, MDD, and substance abuse than males without BPD. |
| Borderline personality subcategories | Andrulonis, P. A. | USA | 1982 | Clinical | 48 | 58 | 106 | Not stated | Identify if there are gender differences in the distinct subcategories of BPD. | Females with BPD are more likely to display interpersonal difficulties, impulsivity, past psychotic episodes, depressive symptoms, eating disorders, antisocial behaviours, family history of depression, psychotic medication and higher frequency of medication than males with BPD. Males with BPD are more likely to act out and to have conduct issues. |
| Brain mechanisms underlying reactive aggression in borderline personality disorder—Sex matters | Herpertz, S. C. | Germany | 2017 | Clinical | 23 | 33 | 56 | 28.02 | Investigate the processing of 1) angry feelings in the context of social rejection and 2) anger-based reactive aggressive behaviour in female and male BPD patients, and 3) explicitly differentiate between a failure in emotion regulation and a failure in behavioural control in states of emotional arousal in BPD. | Males with BPD reported greater aggression and impulsivity than females with BPD. Males showed higher activation in a left-side cluster comprising the hippocampus, amygdala, precuneus, left temporal pole, and in clusters of the medial PFC. |
| Child Maltreatment and Psychiatric Disorders Increase Risk for Stalking Victimization | Bonagura, A. G. | USA | 2022 | Community | 437 | 455 | 892 | Not stated | To determine whether individuals with documented histories of childhood maltreatment and those with psychiatric disorders are at increased risk for stalking victimization. | Both males and females with BPD are at increased risk of lifetime intimate partner stalking victimization, suggesting relationship difficulties that may lead to increased stalking risk. |
| Comorbidity in a clinical sample of substance abusers | Skinstad, A. H. | USA | 2001 | SUD | 125 | 0 | 125 | 29.48 | Examine the prevalence of comorbid psychiatric disorder in a group of male substance dependent subjects. | Males with BPD more likely to have comorbid depression, anxiety disorders, and substance abuse. |
| DBT in an outpatient forensic setting | van den Bosch, L. M. C. | Netherlands | 2012 | Forensic | 10 | 19 | 29 | 34.41 | Describe the sociodemographic, clinical, treatment process, and treatment data from an open study among male and female forensic patients with BPD and compare data with those of BPD patients in a non-forensic mental health setting. | Females with BPD reported more suicide attempts and met more BPD criteria. Males with BPD reported more substance abuse and antisocial personality disorder than females. 30% of males and 21% of females dropped out of DBT. |
| Defense Mechanisms and Psychological Characteristics According to Suicide Attempts in Patients with Borderline Personality Disorder | Lee, Y. J. | Korea | 2020 | Clinical | 55 | 70 | 125 | 28.39 | To investigate psychological, symptomatic, and personality characteristics including defence mechanisms in suicide attempters and non-suicide attempters among patients with BPD. | Females with BPD were more likely to attempt suicide than males. |
| Defense styles, hostility, and psychological risk factors in male patients with personality disorders | Paris, J. | Canada | 1996 | Clinical | 61 | 0 | 61 | 30.6 | Compare ratings of defence styles and hostility in male patients with personality disorders, to determine whether either of these is related to a BPD or to specific psychological risk factors. | Males with BPD reported more unstable relationship, higher hostility, and more maladaptive behaviours than males without BPD. |
| Developmental trajectories to male borderline personality disorder | Goodman, M. | USA | 2013 | Clinical | 97 | 0 | 97 | 25.8 | Identify precursors of BPD in males through parent-reported surveys about their BPD male offspring and non-BPD male siblings. | Males with BPD had various BPD symptoms and more likely to experience early abuse than males without BPD. |
| Dialectical behavior therapy for men with borderline personality disorder and antisocial behavior: A clinical trial | Wetterborg, D. | Sweden | 2020 | Clinical | 30 | 0 | 30 | 35.3 | Investigate the utility of implementing a 12-month DBT program for males with BPD and antisocial behaviour, delivered in addition to usual care at two adult psychiatric outpatient units. | Males with BPD reported reduction in self-harm and depressive symptoms after DBT and were satisfied with the therapy. Dropout rate was 29.7%. |
| Dialectical behaviour therapy (DBT) for forensic psychiatric patients: An Italian pilot study | Bianchini, V. | Italy | 2019 | Clinical | 0 | 19 | 19 | Not stated | Evaluate DBT with a group of offender‐patients in the Italian high intensity therapeutic facilities. | Males with BPD tend to have a history of substance abuse. DBT was found to reduce impulsivity. |
| Discriminating borderline from antisocial personality disorder in male patients based on psychopathology patterns and type of hostility | Hatzitaskos, P. K. | Greece | 1997 | Clinical | 41 | 0 | 41 | 23 | Assess the differences in psychopathology and hostility patterns between patients with BPD or antisocial personality disorder. | Males with BPD reported higher introverted hostility and lower extroverted hostility and had higher scores for depression compared to males without BPD. |
| Dissociative symptoms and self-reported childhood and current trauma in male incarcerated people with borderline personality disorder - results from a small cross-sectional study in Iran | Golshani, S. | Iran | 2020 | Forensic | 69 | 0 | 69 | 33.72 | To understand the association between childhood, adulthood trauma, and dissociative symptoms among male incarcerated people with BPD. | Both childhood and adulthood trauma are highly prevalent among male incarcerated people with BPD. The occurrence of childhood trauma was significantly higher than normative data. |
| Dyadic emotion regulation in women with borderline personality disorder | Miano, A. | Germany | 2021 | Community | 0 | 30 | 64 | 60.27 | To investigate dyadic emotional regulation (i.e., support seeking and the establishment of closeness) in BPD in reaction to different emotional stressors. | Women with BPD reported a larger increase in negative emotions after the relationship threatening conversation and engaged in more support seeking than HC women. There were also more fluctuations than controls between creating closeness and distance in the personally-threatening situation. |
| Effects of serotonin-2A receptor binding and gender on personality traits and suicidal behavior in borderline personality disorder | Soloff, P. H. | USA | 2014 | Clinical | 20 | 13 | 33 | 27.5 | Examine whether gender has a significant effect on the relationships among serotonin-2A binding, personality traits and suicidal behaviour in BPD. | Females with BPD had significantly greater cerebellum distribution volume than BPD males. Among females with BPD, trait impulsiveness was negatively related to the potential binding values in medial frontal cortex and right medial frontal cortex, while no significant relationships for males. |
| Emotional reactivity of partner violent men with personality disorder during conflict | Trahan, L. H. | USA | 2019 | Clinical | 23 | 0 | 23 | 34.78 | Assess negative affect, psychophysiological reactivity, and antecedents to psychological aggression of males with BPD relative to antisocial personality disorder and no diagnosis. | Males with BPD exhibited less frequent distress and longer periods of anger compared to males without BPD. |
| Enhanced sensitivity and response bias for male anger in women with borderline personality disorder | Veague, H. B. | USA | 2014 | Clinical | 0 | 15 | 15 | 27.8 | Examine the association between BPD and early childhood adversity, and the link between BPD and sensitivity to rejection. | Females with BPD respond slower to facial emotions and are more likely to have comorbid obsessive-compulsive personality disorder compared to females without BPD. |
| Examining sex differences in DSM-IV borderline personality disorder symptom expression using Item Response Theory (IRT) | Hoertel, N. | USA | 2014 | Clinical | 386 | 644 | 1030 | 40.32 | Examine whether there are gender differences in the likelihood of reporting DSM-IV BPD symptoms. | Males with BPD reported higher impulsivity and paranoid ideation while females with BPD report higher suicide, affective instability, and chronic emptiness. |
| Experiences of care by Australians with a diagnosis of borderline personality disorder | Lawn, S. | Australia | 2015 | Clinical | 18 | 129 | 147 | No age limit | Explore the experiences of care from the perspective of individuals with BPD. | Males with BPD less likely to seek treatment than females with BPD. |
| Explicit and Implicit Measures of Identity Diffusion in Adolescent Girls With Borderline Personality Disorder | Plakolm Erlač, S. | Solvenia | 2022 | Clinical | 0 | 30 | 63 | 16.29 | To comprehensively assess identity development in adolescents, specifically to assess borderline features and identity diffusion. | Adolescent girls with BPD differ from healthy individuals not only in their conscious representation but also in their implicit representation of the self with regard to BPD related characteristics. |
| Factors associated with completed suicide in borderline personality disorder | Kullgren, G. | Sweden | 1988 | Clinical | 14 | 14 | 28 | 34.11 | Examine the risk factors for completed suicides in BPD. | Males with BPD who attempted suicide showed a more extensive suicidal behaviour at admission than did their matched control subjects. |
| Frequency of borderline personality disorder among psychiatric outpatients in Shanghai | Wang, L. | China | 2012 | Clinical | 48 | 130 | 178 | Not stated | Investigate the frequency, clinical characteristics, and comorbidity of BPD among psychiatric outpatients in two clinics at Shanghai Mental Health Center. | The frequency of BPD among the psychiatric outpatients was 5.8%, with a frequency of 3.5% among males and 7.5% among females. Males with BPD more likely to have comorbid narcissistic personality disorder, obsessive-compulsive personality disorder, and paranoid personality disorder than females with BPD. |
| Frequency of borderline personality disorder in a sample of French high school students | Chabrol, H. | France | 2001 | Clinical | 4 | 18 | 22 | 16.7 | Estimate the frequency of BPD in French high school students. | Overall prevalence of BPD was 10% for boys and 18% for girls. |
| Gender determines cortisol and alpha-amylase responses to acute physical and psychosocial stress in patients with borderline personality disorder | Inoue, A. | Japan | 2015 | Clinical | 39 | 33 | 72 | 23.3 | Examine the psychological, physiological, and neuroendocrine responses to physical and psychosocial stressor in female and male BPD patients compared with healthy participants. | Following Trier Social Stress Test exposure, salivary cortisol levels significantly decreased in females and significantly increased in males when compared with controls. |
| Gender differences and similarities in aggression, suicidal behaviour, and psychiatric comorbidity in borderline personality disorder | Sher, L. | USA | 2019 | Clinical | 145 | 203 | 348 | 34.18 | Examine gender differences and similarities in aggression, impulsivity, suicidal behaviour, and psychiatric comorbidity in BPD compared with healthy controls. | Males with BPD had higher impulsivity and aggression scores than females. Males with BPD were more likely to have comorbid narcissistic, antisocial, paranoid, and schizotypal personality disorders, alcohol and substance use disorders but less likely to have dependent and obsessive-compulsive personality disorders compared to females with BPD. |
| Gender differences in a clinical sample of patients with borderline personality disorder | Banzhaf, A. | Germany | 2012 | Clinical | 57 | 114 | 171 | Not stated | Investigate gender differences and similarities in patients with BPD with respect to Axis I comorbidity, Axis II comorbidity, general psychopathology, and dimensional personality traits. | Males with BPD more often fulfilled the diagnostic criteria for binge eating disorder, antisocial personality disorder, narcissistic personality disorder, and conduct disorder in childhood, whereas females had higher frequencies of bulimia nervosa, posttraumatic stress disorder, and panic disorder with agoraphobia. |
| Gender differences in Axis I and Axis II comorbidity in patients with borderline personality disorder | Tadić, A. | Germany | 2009 | Clinical | 49 | 110 | 159 | 33 | Investigate gender differences in Axis I and Axis II comorbidity and in diagnostic criteria in BPD patients. | Males with BPD more often displayed a substance use disorder and had a higher frequency of antisocial personality disorder. On the other hand, females more frequently had an affective, anxiety or eating disorder. Regarding the BPD diagnostic criteria, males more often displayed intensive anger, whereas women more frequently showed affective instability. |
| Gender differences in borderline personality disorder: Findings from the collaborative longitudinal personality disorders study | Johnson, D. M. | USA | 2003 | Clinical | 65 | 175 | 240 | 31.88 | Examine gender differences in BPD. | Males with BPD more likely to have substance use disorders, and comorbid schizotypal, narcissistic, and antisocial personality disorders. Females with BPD more likely experience identity disturbance and to present with comorbid post-traumatic stress disorder and eating disorders. |
| Gender differences in borderline personality disorder: Results from a multinational, clinical trial sample | Silberschmidt, A. | USA | 2015 | Clinical | 211 | 559 | 770 | 32.4 | Extend previous research by considering gender differences in BPD using both dimensional self-reported and clinical measures of symptomatology. | Females with BPD have greater hostility and relationship disruption than males. Females show greater prevalence of eating disorders and overall symptomatology, including depressive, anxious, and somatic symptoms. Men have higher rates of antisocial personality disorder. |
| Gender differences in sexual preference and substance abuse of inpatients with borderline personality disorder | Dulit, R. A. | USA | 1993 | Clinical | 27 | 110 | 137 | 28.93 | Examine gender differences in a large and diversified sample of borderline inpatients in an acute general psychiatric hospital. | Males with BPD more likely to receive multiple substance use diagnoses, especially of combinations of alcohol, cocaine, and stimulants. |
| Gender Differences in the Treatment of Patients With Borderline Personality Disorder | Dehlbom, P. | Sweden | 2021 | Clinical | 802 | 4728 | 5530 | 34.65 | To investigate gender differences in treatment and the overall mental health care utilization of patients with BPD. | Few men are diagnosed with BPD and those who are diagnosed are likely to receive somewhat less psychiatric medication and psychological therapies compared to women. |
| Grandiose and vulnerable narcissism in borderline personality disorder | Euler, S. | Switzerland | 2018 | Clinical | 15 | 50 | 65 | 29.4 | Explore the associations between total, grandiose, and vulnerable narcissism and gender, diagnostic features of BPD and narcissistic personality disorder, and psychopathology in BPD. | Male patients with BPD displayed higher narcissistic scores than females. |
| Hippocampal volume reduction and history of aggressive behaviour in patients with borderline personality disorder | Zetzsche, T. | Germany | 2007 | Clinical | 0 | 25 | 25 | 26.1 | 1) Confirm previous findings of hippocampal volume reduction in a large sample of BPD patients. 2) Investigate if hippocampal volume loss is correlated with an increase of impulsivity and aggression in BPD patients. | Females with BPD have significant hippocampal volume reduction on the right side versus females without BPD. |
| Homocysteine as a potential indicator of endothelial dysfunction and cardiovascular risk in female patients with borderline personality disorder | Kern, K. | Germany | 2022 | Clinical | 0 | 49 | 99 | 23.91 | To examine homocysteine in BPD and to explore its association with common risk factors for cardiovascular diseases as well as with childhood adversity, chronic stress, and quality of sleep. | BPD patients showed significantly higher mean plasma homocysteine concentrations compared to controls. This correlated significantly with the severity of childhood trauma, chronic stress, and subjective sleep disturbances. |
| Hospitalization and pharmacotherapy for borderline personality disorder in a psychiatric emergency service | Pascual, J. C. | Spain | 2007 | Clinical | 379 | 653 | 1032 | 31.3 | Investigate factors associated with clinicians' decisions to hospitalize patients with borderline personality disorder after they receive psychiatric emergency services or to prescribe psychotropics. | Males with BPD more likely to be prescribed with antipsychotic medications than females with BPD. |
| Identifying a borderline personality disorder prodrome: Implications for community screening | Stepp, S. D. | USA | 2017 | Community | 0 | 299 | 299 | Not stated | Identify an early prodrome of BPD that can be detected from parent and teacher ratings of child temperament and psychopathology dimensions to aid in early intervention. | For females with BPD, parent and teacher ratings of emotionality and sociability, inattention, hyperactivity/ impulsivity and depression severity, as well as parent-reported oppositional behaviour and anxiety predicted the onset of BPD. |
| Impulsivity, gender, and response to fenfluramine challenge in borderline personality disorder | Soloff, P. H. | USA | 2003 | Clinical | 20 | 44 | 64 | 28.4 | To examine the prolactin response to fenfluramine challenge in both male and female subjects with criteria-defined BPD. | Only male and not female participants with BPD had significantly diminished prolactin responses compared to controls Males had higher impulsivity and aggression than females. |
| Increased testosterone levels and cortisol awakening responses in patients with borderline personality disorder: Gender and trait aggressiveness matter | Rausch, J. | Germany | 2015 | Clinical | 20 | 35 | 55 | 27.4 | Investigate alterations in saliva testosterone and cortisol levels in female and male patients with BPD compared to healthy controls. | Females with BPD showed increased and steeper cortisol awakening response compared to males with BPD. |
| Loudness dependence of auditory evoked potentials in patients with borderline personality disorder-Impact of psychopathology | Schaaff, N. | Germany | 2012 | Clinical | 0 | 9 | 9 | 22.44 | Compare BPD to matched healthy subjects on psychopathological characteristics and investigate correlations of neurophysiological data with psychopathological dimensions. | Females with BPD had significantly higher impulsiveness, anger, and co-occurring depressive symptoms than females without. |
| Men and women with borderline personality disorder resident in Dutch special psychiatric units in prisons: A descriptive and comparative study | van den Brink, C. | Netherlands | 2018 | Forensic | 106 | 61 | 167 | 35.8 | Describe the characteristics of BPD among offender patients and test for gender differences in abuse and/or neglect in childhood, Axes I and/or II comorbidities, and presenting clinical symptoms. | Females with BPD had significantly higher on anxiety, depression, psychosis, negative symptoms, and activation than males. Males more likely to have comorbid substance use disorder. |
| Negative bias and reduced visual information processing of socio-emotional context in Borderline Personality Disorder: A support for the hypersensitivity hypothesis | Bortolla, R. | Italy | 2020 | Mixed | 0 | 20 | 40 | 23.23 | To investigate the role of negative bias and avoidance mechanisms in BPD. | Findings provide evidence on dysfunctional mechanisms sustaining emotional dysregulation in BPD. |
| Network Analysis of DSM Symptoms of Substance Use Disorders and Frequently Co-Occurring Mental Disorders in Patients with Substance Use Disorder Who Seek Treatment | López-Toro, E. | Mixed | 2022 | SUD | 131 | 62 | 772 | 9.44 | To examine the interrelationships between DSM symptoms of SUD and four frequently co-occurring mental disorders (ADHD, conduct disorder (CD), major depressive disorder (MDD), and BPD) in women and men seeking SUD treatment. | BPD was significantly more prevalent in female compared to male patients. |
| Neural correlates of emotional action control in anger-prone women with borderline personality disorder | Bertsch, K. | Germany | 2018 | Clinical | 0 | 30 | 30 | 26.9 | Investigate if similar reductions in the communication between the prefrontal cortex and amygdala during emotional action control could be found in anger-prone women with BPD, and whether alterations are related to elevated levels of endogenous testosterone and tendency to act out. | Females with BPD had higher outwardly directed anger and faster approach versus avoidance responses to angry faces compared to females without BPD. Females with BPD had higher depressiveness, trait anxiety, attention deficit hyperactivity disorder symptoms and testosterone levels compared to females without BPD. |
| Out of control? Acting out anger is associated with deficient prefrontal emotional action control in male patients with borderline personality disorder | Bertsch, K. | Germany | 2019 | Not stated | 15 | 0 | 15 | 28.3 | Investigate the involvement of lateral antero- and dorsal prefrontal cortex in the control of fast emotional actions and its relation to self-reported tendencies to act out anger. | Males with BPD had significantly higher impulsivity, depression, trait anxiety, attention deficit hyperactivity disorder, anger-out, and BPD symptom severity compared with healthy controls. Males with BPD also showed reduced anterolateral prefrontal activations during emotional action control compared to controls. |
| Outcomes in women diagnosed with borderline personality disorder in adolescence | Biskin, R. S. | Canada | 2011 | Clinical | 0 | 47 | 47 | 19.55 | Explore the longer-term outcomes of females diagnosed with BPD in adolescence, and factors that might be associated with these outcomes. | Females with BPD who did not remit were significantly more likely to have a current episode of MDD, lifetime substance use disorder, and self-reported childhood sexual abuse. |
| Persistent antinociception through repeated self-injury in patients with borderline personality disorder | Magerl, W. | Germany | 2012 | Clinical | 7 | 15 | 22 | 29.5 | Analyse the relationship of pain perception to self-injurious behaviour and probe possible gender differences by including a small subgroup of male BPD patients. | Males with BPD scored significantly lower on interpersonal functioning and higher on ASPD than females. In contrast, females have higher identity diffusion, more recent self-injurious behaviour, and higher pain thresholds than males. |
| Personality disorder prevalence and correlates in a whole of nation dataset | Newton-Howes, G. | New Zealand | 2021 | Clinical | Not stated | Not stated | 294612 | Not stated | To describe:  1) the prevalence of personality disorder among those being treated by secondary mental health services in New Zealand, overall and specifically for Māori. 2) This population in terms of demographic and clinical factors, by personality disorder type, and gender.  3) Annual service use patterns for those with diagnosis of personality disorder, in comparison to those with a diagnosis of depression. | Borderline personality disorder was the most common specific diagnosis for women; while for men, antisocial personality disorder was the most common followed by borderline personality. |
| Personality disorders among patients accessing alcohol detoxification treatment: prevalence and gender differences | Picci, R. L. | Italy | 2012 | SUD | 38 | 24 | 62 | Not stated | Investigate personality disorders among patients accessing inpatient alcohol detoxification treatments and to describe gender differences. | Females with BPD more likely to be divorced, educated, and older when they abused alcohol than males. |
| Predicting outcome for borderline personality disorder | Links, P. S. | Canada | 1990 | Clinical | 22 | 47 | 69 | 25.3 | Examine the psychosocial outcome of borderline patients at 2 years’ follow-up and to determine whether the proposed variables are predictive of the short-term, as well as long-term, outcome. | Males with BPD more likely to drop out of treatment than females. |
| Predicting psychotherapy utilization for patients with borderline personality disorder | Löffler-Stastka, H. | Austria | 2003 | Clinical | 10 | 10 | 20 | 38.3 | Determine which patients engaged in further outpatient psychotherapy after a 6-week voluntary inpatient therapy with a psychoanalytic orientation provided in a social-psychiatric care unit in a mental health hospital. | Females with BPD more likely to engage in further psychotherapy than males. |
| Predictors of self-mutilation in patients with borderline personality disorder: A 10-year follow-up study | Zanarini, M. C. | USA | 2011 | Clinical | 57 | 233 | 290 | 26.9 | Assess the predictors of self-mutilation over a decade of prospective follow-up in a large sample of diagnosed patients with BPD. | Female gender was a predictor of self-mutilation across the five follow-up periods. |
| Preliminary outcomes on the use of dialectical behavior therapy to reduce hospitalization among adolescents in residential care | Sunseri, P. A. | USA | 2004 | Clinical | 0 | 68 | 68 | 14.46 | Examine if DBT can help to decrease the number of premature discharges due to suicidality, reduce the number of days spent in psychiatric hospitals, and reduce the duration of client restraints and seclusions. | DBT for females with BPD was effective in achieving a significant reduction in both premature terminations due to suicidality, and in the number of days clients spent in psychiatric hospitals due to self-injurious behaviours. |
| Prevalence of the metabolic syndrome in patients with borderline personality disorder: Results from a cross-sectional study | Kahl, K. G. | Germany | 2013 | Clinical | 14 | 121 | 135 | 31.9 | Examine the prevalence of the metabolic syndrome and its components in BPD and to compare the obtained data with those from primary care subjects of the same region. | Females with BPD have higher abdominal obesity and hypertriglyceridemia, and lower fasting glucose than males. |
| Prevalence rates of personality disorder and its association with methamphetamine dependence in compulsory treatment facilities in China | Zhang, C. | China | 2018 | SUD | 106 | 0 | 106 | Not stated | Investigate the prevalent rates of personality disorders in methamphetamine users and further explore the association between personality disorder and methamphetamine dependence. | BPD for males was a risk factor of methamphetamine dependence. |
| Prior head injury in male veterans with borderline personality disorder | Streeter, C. C. | USA | 1995 | Clinical | 43 | 0 | 43 | 32 | Assess the relationship of head injury to BPD in male veterans. | BPD for males was associated with changes of substance abuse, learning disability, conduct issues, brain seizures, and brain trauma. |
| Proactive and reactive violence among intimate partner violent men diagnosed with antisocial and borderline personality disorder | Ross, J. M. | USA | 2009 | Clinical | 23 | 0 | 23 | 35 | Examine severity of intimate partner violent and the use of proactive versus reactive violence among men diagnosed with antisocial personality disorder, BPD, and controls. | Males with BPD appear to use violence more reactively. |
| Psychobiological Correlates of Aggression in Female Adolescents with Borderline Personality Disorder | Cavelti, M. | Germany | 2022 | Mixed | 0 | 21 | 46 | 15.28 | To examine aggressive behaviour and its biological correlates in adolescents with BPD compared to healthy controls. | Findings suggests that adolescents with BPD experienced fearful stress in anticipation of the experimental task in contrast to healthy controls who showed an adaptive response of the autonomic nervous system necessary to deal with the upcoming demand. |
| Psychopathy scores reveal heterogeneity among patients with borderline personality disorder | Newhill, C. E. | USA | 2010 | Clinical | 103 | 118 | 221 | 29.24 | Investigate the nature of the underlying heterogeneity in BPD in relation to psychopathy to provide a better understanding of subgroups of individuals that meet criteria for BPD but present differences in terms of other psychiatric symptoms and antisocial behaviours. | Females with BPD comprised a significantly greater proportion of low psychopathic subgroup. Males with BPD comprised a significantly greater proportion of high psychopathic/ antisocial subgroup. Females tend to stay longer in treatment than males. |
| Race and sexually transmitted diseases in women with and without borderline personality disorder | De Genna, N. M. | USA | 2011 | Clinical | 0 | 113 | 113 | 27.14 | Examine the history of sexually transmitted diseases among females with BPD with and without a lifetime substance use disorder, and to compare their histories to those of a group of women with a current nonpsychotic axis I disorder. | Females with BPD more likely to be at risk of sexually transmitted diseases, post-traumatic stress disorder, panic disorder, but less likely to have MDD compared to females without BPD. |
| Recidivistic offending and mortality in alcoholic violent offenders: A prospective follow-up study | Tikkanen, R. | Finland | 2009 | SUD | 242 | 0 | 242 | 32.4 | Examine BPD and negative childhood circumstances as hypothesized predictors of important long-term outcome on recidivistic offending and mortality. | BPD was one of the risk factors for recidivistic violence in males. A combination of BPD and childhood maltreatment was particularly concerning, suggesting an additive risk increase for a poor outcome. |
| Relationship between Suicide Attempt History and Borderline Personality Disorder, Aggression, Impulsivity, and Self-Mutilative Behavior among Male Inpatients with Substance Use Disorder | Karabulut, V. | Turkey | 2021 | SUD | 80 | 0 | 132 | 37.22 | To evaluate the relationship between lifetime history of suicide attempt and BPD, aggression, impulsivity, and self-mutilative behaviour in a sample with SUD. | Those with history of suicide attempt had a significantly higher rate of BPD diagnosis. However, BPD was no longer a predictor when self-mutilating behaviour was included in the analysis. |
| Relationship of sex to symptom severity, psychiatric comorbidity, and health care utilization in 163 subjects with borderline personality disorder | McCormick, B. | USA | 2007 | Clinical | 25 | 138 | 163 | 31.03 | Examine the relationship between sex, symptom severity, patterns of psychiatric comorbidity, quality of life variables, and health care utilization in these subjects. | Females with BPD more likely than males to have an anxiety disorder, somatoform disorders, and histrionic personality disorder. Females also had higher ratings of depression, anxiety, obsessive-compulsiveness, work dysfunction, and negative affectivity, and were more likely to endorse the “paranoia/dissociation” BPD criterion. In contrast, antisocial personality disorder was more common in males. |
| Relationship quality and stability in couples when one partner suffers from borderline personality disorder | Bouchard, S. | Canada | 2009 | Clinical | 0 | 35 | 35 | 33.83 | Explore attachment status, communication style, intimate violence, and dyadic adjustment in couples where the women suffer from BPD and couples from a nonclinical control group. | Females with BPD have greater avoidance of intimacy, more dissatisfied with relationship, exhibit more violence than females without BPD. Females with BPD also experienced more violence. |
| Reported pathological childhood experiences associated with the development of borderline personality disorder | Zanarini, M. C. | USA | 1997 | Clinical | 82 | 276 | 358 | 27.6 | Examine the role of childhood sexual abuse in the etiology of BPD in perspective by studying a wide range of pathological childhood experiences. | Female gender was found to be a significant predictor of a borderline diagnosis. |
| Risk factors for borderline personality in male outpatients | Paris, J. | USA | 1994 | Clinical | 61 | 0 | 61 | 30.6 | Examine the role of several psychological risk factors (childhood sexual abuse and its parameters, childhood physical abuse and its parameters, early separation or loss, and abnormal parenting bonding) in male patients with BPD. | Males with BPD had higher frequency and more severe child sexual abuse, longer duration of physical abuse, increased rates of early separation or loss, and a higher paternal control. Trauma and loss, as well as problems with fathers, are important for the development of BPD in males. |
| Rorschach responses in Borderline Personality Disorder with alcohol dependence | Skinstad, A. H. | Norway | 1999 | SUD | 29 | 0 | 29 | 32.3 | Analyse differences in Rorschach responses between two groups of alcoholics, one of which was diagnosed BPD and the other Mixed Personality Disorder. | Males with BPD were found to be more interested in other people, more impulsive, more aggressive, and more likely to withdraw from social interactions than males without BPD. |
| Sex and ethnic distribution of borderline personality disorder in an inpatient sample | Castaneda, R. | USA | 1985 | Clinical | 32 | 69 | 101 | 21.5 | Examine the sex and ethnic distribution of BPD in a psychiatric inpatient sample. | Fewer men than women were diagnosed as having BPD among whites and blacks but not among Hispanics. |
| Sexual attitudes and activities in women with borderline personality disorder involved in romantic relationships | Bouchard, S. | Canada | 2009 | Clinical | 0 | 34 | 34 | 33.47 | 1) Explore the association between the sexual attitudes of women with BPD and their sexual activities in comparison to a matched sample of women from the community. 2) Assess whether sexual attitudes are associated with attachment representations, the frequency of sexual intercourse, and their marital or couple satisfaction. | Females with BPD tend to have more sexual partners, more psychological distress, higher avoidance, more negative attitude towards sex, higher anxiety, higher insecure attachment, and higher sexual abuse during childhood than females without BPD. |
| Sexual practices among patients with borderline personality disorder | Zubenko, G. S. | USA | 1987 | Clinical | 19 | 61 | 80 | 24.8 | Replicate and extend the findings that male patients with BPD are predominantly or exclusively homosexual. | Homosexuality was 10 times more common among the men and six times more common among the women with BPD than in the general population or in a depressed control group. |
| Sexuality and sexual health among female youth with borderline personality disorder pathology | Thompson, K. N. | Australia | 2019 | Clinical | 0 | 50 | 50 | Not stated | Investigate aspects of the sexuality and sexual health among female youths with BPD pathology, and to compare this with a matched healthy population sample. | Females with BPD engaged in sexual relationships at a younger age with more sexual partners and casual relationships than females without BPD. They were more likely to practice unsafe sex for their first sexual experience, to be coerced into unwanted sexual activity, to be unclear about their sexual identity or their sexual attraction, and to report worse overall health status. |
| Social role dysfunction and coping in borderline personality disorder | Carlson, E. M. | Canada | 2020 | Clinical | 73 | 160 | 233 | 37.32 | To examine gender’s moderating effect on the association between BPD symptoms and social maladjustment through coping. | As BPD symptoms increased among women, emotion-oriented coping also increased, which in turn was related to an increase in social role dysfunction, encompassing difficulties performing in norm-informed, socially sanctioned ways in roles such as employee or partner. |
| SSRI treatment of borderline personality disorder: A randomized, placebo-controlled clinical trial for female patients with borderline personality disorder | Rinne, T. | Netherlands | 2002 | Clinical | 0 | 38 | 38 | 29.2 | Investigate the effects of fluvoxamine on the borderline symptoms of rapid mood shifts, impulsivity, and anger. | Fluvoxamine but not placebo produced a robust and long-lasting reduction in the scores on the subscale for rapid mood shifts for females with BPD. |
| Striatal activity in borderline personality disorder with comorbid intermittent explosive disorder: Sex differences | Perez-Rodriguez, M. M. | USA | 2012 | Clinical | 22 | 16 | 38 | 30.5 | Compare volume and striatal activity in a group of BPD patients selected for serious impulsive aggression and healthy controls in an aggression provocation behavioural paradigm. | Males with BPD had significantly lower striatal relative glucose metabolism than all other groups. These sex differences suggest differential involvement of frontal-striatal circuits in BPD and are involved in affective learning and social decision-making. |
| Structural brain abnormalities in borderline personality disorder: A voxel-based morphometry study | Soloff, P. | USA | 2008 | Clinical | 12 | 22 | 34 | 27.5 | Use voxel-based morphometry as an exploratory technique to study brain-behaviour relationships of diagnosis, gender, childhood sexual abuse, depressed mood, impulsivity and aggression on group differences. | Females with BPD but not males, had significant reductions in medial temporal lobe, including the amygdala. Males with BPD but not females, showed diminished grey matter concentrations in the anterior cingulate gyrus compared with findings from healthy controls. |
| Substance abuse in borderline personality disorder | Miller, F. T. | USA | 1993 | Clinical | 5 | 35 | 40 | 29 | Document the impact of comorbid substance abuse on the patients’ social and economic adjustment and investigate the association of substance use with symptoms highly correlated with BPD. | Female with BPD preferred alcohol and sedatives. Male with BPD preferred stimulants. |
| Substance abuse patterns and their association with psychopathology and type of hostility in male patients with borderline and antisocial personality disorder | Hatzitaskos, P. | Greece | 1999 | Clinical | 41 | 0 | 41 | 23 | 1) Investigate the prevalence of substance use disorder in young adult patients with BPD. 2) Assess whether alcohol and drug abuse in the patients related to their psychopathology and hostility. | Males with BPD less likely to abuse substances compared to males with antisocial personality disorder, and the use is negatively associated with depression. |
| Temperament and character in patients with borderline personality disorder taking gender and comorbidity into account | Barnow, S. | Germany | 2007 | Clinical | 66 | 136 | 202 | 30.1 | 1) Examine the degree of harm avoidance, novelty seeking, self-directedness and cooperativeness in inpatients with BPD compared to psychiatrically healthy individuals. 2) Examine correlations of BPD symptoms with scales from Cloninger’s psychobiological model taking gender and psychiatric comorbidity into consideration. | Males with BPD presented simultaneously high levels of novelty seeking and harm avoidance. In contrast, females with BPD were characterized by high levels of harm avoidance, but not novelty seeking. |
| The impact of borderline personality disorder on residential substance abuse treatment dropout among men | Tull, M. T. | USA | 2012 | SUD | 34 | 0 | 34 | Not stated | 1) Examine the impact of BPD on residential substance abuse treatment dropout within a sample of male substance use disorder patients. 2. Examine the extent to which BPD is differentially associated with different types of treatment dropout. | Males with BPD were significantly more likely to prematurely dropout of treatment. Males with BPD were also more likely to experience centre-initiated dropout as opposed to voluntary withdrawal from treatment. |
| The quality of depression in adolescents with borderline personality disorder | Wixom, J. | USA | 1993 | Clinical | 0 | 35 | 35 | 15.6 | Examine whether the quality of depressive experiences in borderline patients can be distinguished from that of other depressed, non-borderline patients. | Males with BPD reported higher dependency and self-criticism compared to those without, substance abuse, and antisocial personality disorder. |
| The relationship between adverse childhood experiences, emotional states and personality disorders in offenders | Koolen, R. | Netherlands | 2022 | Forensic | 102 | 0 | 102 | 38.3 | 1) To examine whether schema modes distinguish and predict these personality disorders 2) To examine the schema therapy theory regarding the relationship between different types of adverse childhood experiences and personality disorders, by testing whether schema mode factors mediate the relationship between adverse childhood experiences and personality disorders in an offender sample. | ASPD appears to be characterized by more externalizing emotional states and BPD by more internalized emotional states. |
| The relationship between childhood traumas, dissociation, and impulsivity in patients with borderline personality disorder comorbid with ADHD | Kulacaoglu, F. | Turkey | 2017 | Clinical | 37 | 128 | 165 | 22.46 | Determine the complex relationship between trauma, impulsivity, dissociative symptoms, and the impact of attention deficit hyperactivity disorder on this relationship in a sample of patients with BPD. | Being female was a predictor of attention deficit hyperactivity disorder and dissociation in BPD patients. History of any childhood trauma and dissociative symptoms were found to be higher in female patients. |
| The relationship between illegal behaviors and borderline personality symptoms among internal medicine outpatients | Sansone, R. A. | USA | 2012 | Clinical | 128 | 247 | 375 | 50.34 | Examine if there is a relationship between history of illegal behaviors and BPD in an outpatient internal medicine sample. | Males with higher BPD more likely to engage in a greater number of different illegal behaviors, whereas the reverse pattern was evident among females. |
| The role of gender in the clinical presentation of patients with borderline personality disorder | Zlotnick, C. | USA | 2002 | Clinical | 44 | 105 | 149 | 31.4 | Examine gender differences in comorbidity and in level of impairment among borderline patients. | Males with BPD reported significantly more lifetime substance abuse disorders, antisocial personality and met criteria of intermittent explosive disorder that did not overlap with a diagnosis of BPD. Females with BPD reported significantly more lifetime eating disorders than males with BPD. |
| The Unhappy Mental Health Triad: Comorbid Severe Mental Illnesses, Personality Disorders, and Substance Use Disorders in Prison Populations | Mundt, A. P. | Chile | 2020 | Forensic | 229 | 198 | 427 | Not stated | To assess the prevalence of co-occurring disorders across the three a priori defined diagnostic groups severe mental disorders, PD, and SUD in prison populations. | 216 (50.6%) of sample meet the criteria for BPD. A greater proprtion of males (67.2%) meet the criteria for BPD than females (31.3%). |
| Three-year follow up of women with and without borderline personality disorder: development of Cloninger's character in adolescence | Ha, K. S. | South Korea | 2004 | Clinical | 0 | 48 | 48 | Not stated | Examine the developmental patterns of Cloninger’s biogenetic character traits in subjects with BPD. | Females with BPD had lower directedness, higher on novelty seeking, and higher score on harm avoidance than females without BPD. |
| Treatment of aggression with topiramate in male borderline patients: A double-blind, placebo-controlled study | Nickel, M. K. | Germany | 2005 | Clinical | 22 | 0 | 22 | 29.5 | Assess the efficacy of topiramate monotherapy for the treatment of anger symptoms in male patients with BPD. | Significant changes on state anger, trait anger, anger out, and anger control were observed in the males with BPD treated with topiramate. |
| Treatment utilization by gender in patients with borderline personality disorder | Goodman, M. | USA | 2010 | Clinical | 86 | 409 | 495 | 23.63 | Investigate patterns of treatment utilization in male and female individuals with BPD, using an online parent-reported questionnaire. | Male with BPD were more likely to use drug/alcohol rehabilitation services and received significantly less lifetime psychotherapy and pharmacotherapy than females with BPD. |
| Two-year randomized controlled trial and follow-up of dialectical behavior therapy vs therapy by experts for suicidal behaviors and borderline personality disorder | Linehan, M. M. | USA | 2006 | Clinical | 0 | 52 | 52 | 29 | Evaluate the hypothesis that unique aspects of DBT are more efficacious compared with treatment offered by non-behavioural psychotherapy experts. | DBT for females with BPD was associated with better outcomes in the intent-to-treat analysis than control during treatment and follow-up. Subjects receiving DBT less likely to make a suicide attempt, required less hospitalization, had lower medical risk, and were less likely to drop out of treatment and had fewer psychiatric hospitalizations and psychiatric emergency department visits. |
| Varieties of impulsivity in males with alcohol dependence: The role of Cluster-B personality disorder | Rubio, G. | Spain | 2007 | SUD | 29 | 0 | 29 | 37 | Specificity of behavioural impairments for alcoholics with Cluster-B personality disorders (Borderline and Antisocial subtypes). | Alcohol-dependent males with BPD made more omission errors than males with antisocial personality disorder. |
| Violence in the lives of adult borderline patients | Zanarini, M. C. | USA | 1999 | Clinical | 57 | 233 | 290 | 26.9 | Assess the relationship between such experiences of violence and a variety of potential risk factors of adult violence reported by a sample of criteria-defined borderline patients and axis II controls. | Female borderline patients were significantly more likely than male borderline patients to have been physically and/or sexually assaulted as adults. |
| Violent women: A multicentre study into gender differences in forensic psychiatric patients | de Vogel, V. | Netherlands | 2016 | Clinical | 275 | 275 | 550 | 34.75 | Explore gender differences in psychopathology and incidents during treatment. | BPD was most common in females than males. |

*Note*. USA = United Stated of America, BPD = Borderline Personality Disorder, MDD = Major Depressive Disorder, PTSD = Post-Traumatic Stress Disorder, DBT = Dialectical Behavior Therapy, IPV = Intimate Partner Violence, SUD = Substance Use Disorder
